# Fracture Toughness of Porous Material of LSCF in Bulk and Film Forms

**DOI:** 10.1111/jace.13507

**Published:** 2015-03-20

**Authors:** Zhangwei Chen, Xin Wang, Finn Giuliani, Alan Atkinson

**Affiliations:** ^1^ Department of MaterialsImperial College LondonLondonSW7 2BPUnited Kingdom

## Abstract

Fracture toughness of La_0.6_Sr_0.4_Co_0.2_Fe_0.8_O_3‐δ_ (LSCF) in both bulk and film forms after sintering at 900°C to 1200°C was measured using both single‐edge V‐notched beam (SEVNB) 3‐point bending and Berkovich indentation. FIB/SEM slice‐and‐view observation after indentation revealed the presence of Palmqvist radial crack systems after indentation of the bulk materials. Based on crack length measurements, the fracture toughness of bulk LSCF specimens was determined to be in the range 0.54–0.99 MPa·m^1/2^ (depending on sintering temperature), in good agreement with the SEVNB measurements (0.57–1.13 MPa·m^1/2^). The fracture toughness was approximately linearly dependent on porosity over the range studied. However, experiments on films showed that the generation of observable indentation‐induced cracks was very difficult for films sintered at temperatures below 1200°C. This was interpreted as being the result of the substrate having much higher modulus than these films. Cracks were only detectable in the films sintered at 1200°C and gave an apparent toughness of 0.17 MPa·m^1/2^ using the same analysis as for bulk specimens. This value is much smaller than that for bulk material with the same porosity. The residual thermal expansion mismatch stress measured using XRD was found to be responsible for such a low apparent toughness.

## 1 Introduction

Over the last few decades, perovskite material La_0.6_Sr_0.4_Co_0.2_Fe_0.8_O_3‐δ_ (LSCF) has been widely used for applications in cathodes for intermediate temperature solid oxide fuel cells (IT‐SOFCs)^1^ and oxygen separation membranes,^2^ thanks to its promising mixed ionic–electronic conductivity^3^ and high oxygen surface exchange rate.^4^ The long‐term durability of such applications also relies on LSCF's mechanical properties in addition to its electrochemical properties as for other components.^5^, ^6^, ^7^ Appropriate mechanical properties are highly desired to avoid possible failures such as cracks, delamination, and fractures induced by mechanical stresses arising from both fabrication and operation.^8^ To date, most studies of mechanical properties of LSCF have focused on elastic modulus and strength determination for nominally dense and porous bulk material using conventional macroscopic techniques such as resonance methods and ring‐on‐ring bending.^7^, ^9^, ^10^, ^11^ In our previous work, we studied elastic modulus and hardness of both porous films and bulk material using nanoindentation.^12^ Another key mechanical property is fracture toughness, which characterizes resistance to fracture, has, however, received little attention, particularly for porous materials.

Mode I fracture toughness (*K*_Ic_) is a material prope‐rty which describes the ability of a material containing a preexisting crack to resist further crack propagation leading to fracture. It is therefore an important parameter for assessing fracture failure since the occurrence of cracks and defects is unavoidable during processing, fabrication, or application.^13^ Literature reports concerning indentation fracture toughness of films on substrates are restricted to dense (or nanoporous) thin coatings.^14^, ^15^, ^16^ This study presents measurements of *K*_Ic_ at room temperature for porous LSCF in both bulk and film forms prepared by sintering at 900°C to 1200°C.

## 2 Experimental Procedure

### 2.1 Specimen Preparation

LSCF specimens in both bulk and film forms were studied. Both the LSCF films deposited on fully dense CGO (Ce_0.9_Gd_0.1_O_2‐δ_) substrates and the bulk LSCF in disk and bar shapes were fabricated followed by sintering in air at 900°C to 1200°C, as detailed in.^12^ The as‐sintered films were typically 10 μm thick and crack free with very smooth surfaces.^17^ The SEVNB specimens were prepared in accordance with ASTM E399 Standard.^18^ A notch was machined using a thin diamond saw, followed by cutting with a razor blade sprayed with diamond suspension to obtain a sharp tip radius for the notches (with a typical width of 20–30 μm).

Porosity *p*, elastic modulus *E*, and indentation hardness *H* have been reliably measured in the previous work^12^ for both bulk and films as summarized in Table 1. As also reported in^19^, ^20^ for various materials, the indentation hardness in this study was found to be similar to the hardness which was measured based on the dimensions of the residual imprint and thus was used for the toughness calculation. The Poisson's ratio ν was assumed to be 0.3 9 where required.

**Table 1:** Summary of Bulk and Film Properties^12^

Sintering temperature (°C)	Bulk			Films		
	*p* (vol%)	*E* (GPa)	*H* (GPa)	*p* (vol%)	*E* (GPa)	*H* (GPa)
900	44.9 ± 0.3	34.2 ± 2.1	0.69 ± 0.09	46.9 ± 2.2	32.4 ± 1.2	0.37 ± 0.08
1000	36.3 ± 1.1	44.5 ± 3.2	0.86 ± 0.20	39.7 ± 2.6	48.3 ± 4.6	0.61 ± 0.11
1100	28.7 ± 0.9	80.2 ± 1.9	2.35 ± 0.14	24.1 ± 1.8	90.0 ± 6.4	1.28 ± 0.14
1200	5.2 ± 0.1	174.3 ± 2.8	5.76 ± 0.12	15.2 ± 1.2	121.5 ± 7.2	1.97 ± 0.20

### 2.2 Single‐Edge V‐Notched Beam (SEVNB) 3‐Point Bending Test

Fracture toughness of the bulk bars was first determined using the SEVNB tests. The bars were loaded up to fracture in 3‐point bend mode using a ZwickiLine Z2.5 testing machine (Zwick, Ulm, Germany). Loading data were recorded with a loading rate of 0.01 N/s. *K*_Ic_ was calculated using Eq. (1),^21^

(1) $$\begin{array}{cc} K_{\mathrm{IC}} & =\frac{3FS\sqrt{\pi b}}{2BW^{2}}(1.09-1.735\left(\frac{b}{W}\right)+8.2{\left(\frac{b}{W}\right)}^{2}\\ & \;-14.18{\left(\frac{b}{W}\right)}^{3}+14.57{\left(\frac{b}{W}\right)}^{4}) \end{array}$$

where *F* is the applied load at fracture, *S* is the predefined span length between the two outer supporting points, *B* is the beam thickness, *W* is the beam width, and *b* is the notch depth. In this study, *S *=* *20 mm, *B *=* *3.5 mm, *W *=* *5 mm, *L *=* *25 mm, and *b *=* *2.5 mm.

### 2.3 Indentation Test

Fracture toughness measurements on both bulk specimens and porous films were also conducted using a combined micro/nanoindentation machine (NanoTest; Micromaterials, Wrexham, UK) with a Berkovich diamond tip. Loads of up to 500 mN (nanoindentation) or from 1 to 20 N (microindentation) were applied as needed. Toughness calculation was based on the resulting crack lengths measurement on the specimen surface. Surface and subsurface of the imprints and cracks were investigated using the FIB/SEM slice‐and‐view technique (Helios NanoLab600; FEI, Hillsboro, OR).

Various formulae^22^, ^23^, ^24^ have been proposed to link the fracture toughness to the indenter type, crack geometry, load, and the properties of the materials under test. For indentation with a Berkovich tip, the mode I fracture toughness was obtained using Eq. (2), 24:

(2) $$K_{\mathrm{IC}}=0.016{\left(\frac{a}{l}\right)}^{1/2}{\left(\frac{E}{H}\right)}^{2/3}\frac{P}{C^{3/2}}$$

where *E* and *H* are elastic modulus and hardness as summarized in Table 1. *P* is the maximum indentation load, *c* is the radial crack length measured by SEM from indent center to the radial crack tip (i.e., crack end), *a* is the diagonal length of the indent from center to the indent corner, and *l* is the crack distance measured from the indent corner to the crack tip and is *l = c−a*.

Equation (2) is also thought to be applicable to the toughness measurement of supported films under appropriate conditions. However, the results might be influenced by the substrate, the residual stress, and the permanent pore‐filling deformation when the films are porous. These can significantly complicate the fracture toughness measurement of the film. For example, the effect of the substrate has been acknowledged in some previous work on fully dense coatings,^13^, ^25^ but there has been no in‐depth investigation. Moreover, to the best of our knowledge, no information can be found in the literature regarding such effects on the fracture measurements of porous thin films deposited on hard substrates.

### 2.4 XRD Residual Stress Measurement

The typical average values of the thermal expansion coefficient (TEC) for LSCF and CGO were taken as 15.3 × 10^−6^ and 12.5 × 10^−6^ K^−1^, respectively, according to.^1^, ^26^, ^27^ The existence of such a TEC mismatch will induce tensile equi‐biaxial residual stresses in the films after cooling. Therefore, the determination of the residual stresses in the films is critical for interpreting the fracture experiments. XRD is a reliable technique to determine the residual stresses in thin polycrystalline films.^28^ If the specimen is in a plane equi‐biaxial stress state (σ) which corresponds to linear elastic strain (ε) of the crystal lattice, then the angular position of a chosen diffraction peak will be shifted (by Δ2θ). To increase the measurement precision, diffraction peaks generated at high angles are usually chosen (i.e., 2θ > 120°). If the specimen is tilted by an angle ψ (where ψ is the angle of the normal of the lattice diffraction plane to the specimen surface normal) then it is not necessary to know the strain‐free lattice parameter.

The measurements were carried out at room temperature with the so‐called sin^2^ψ method^28^ using a Philips PW1729 (Philips, Eindhoven, the Netherlands) XRD machine (operated at 40 kV and 40 mA) with a copper target producing Cu*K*_α_ radiation. Scans were conducted at a number of ψ values from 0°, 12.92°, 18.44°, 22.79°, and 26.57°, corresponding to sin^2^ψ values of 0.0–0.2 with an increment of 0.05. The interplanar *d*‐spacing was plotted as a function of sin^2^ψ and Eq. (3), 28 used to calculate the residual stresses in the as‐sintered LSCF thin films.

(3) $$\sigma =KM=\left(\frac{E}{1+\nu }\right)\frac{1}{d_{\psi 0}}\left(\frac{\partial d_{\psi }}{\partial \sin^{2}\psi }\right)$$

where $K=\left(\frac{E}{1+\nu }\right)\frac{1}{d_{\psi 0}}$ is a constant and *E* is the previously measured modulus. ν is the Poisson's ratio which was assumed to be 0.3. *d*_ψ0_ is the measured lattice spacing when ψ_0_ **= 0, and $M=\left(\frac{\partial d_{\psi }}{\partial \sin^{2}\psi }\right)$ is the slope of the plot of *d*‐spacing versus sin^2^ψ.

## 3 Results

### 3.1 SEVNB 3‐Point Bending Test for Bulk Specimens

The fracture toughness measured based on the SEVNB tests is shown in Table 2. *K*_Ic_ increased with increasing sintering temperature as expected.

**Table 2:** Fracture Toughness Measured by SEVNB Tests for Bulk Specimens

Specimen sintering temperature (°C)	900	1000	1100	1200
Fracture toughness at RT (MPa·m^1/2^)	0.57 ± 0.05	0.68 ± 0.07	0.74 ± 0.08	1.13 ± 0.15

### 3.2 Indentation Results for Bulk Specimens

Although the indentation‐induced cracks did not generate identifiable pop‐in events in the indentation load–displacement curves, they were detected in SEM examinations.

#### 3.2.1 Microstructural Characterization

**Figure 1:**
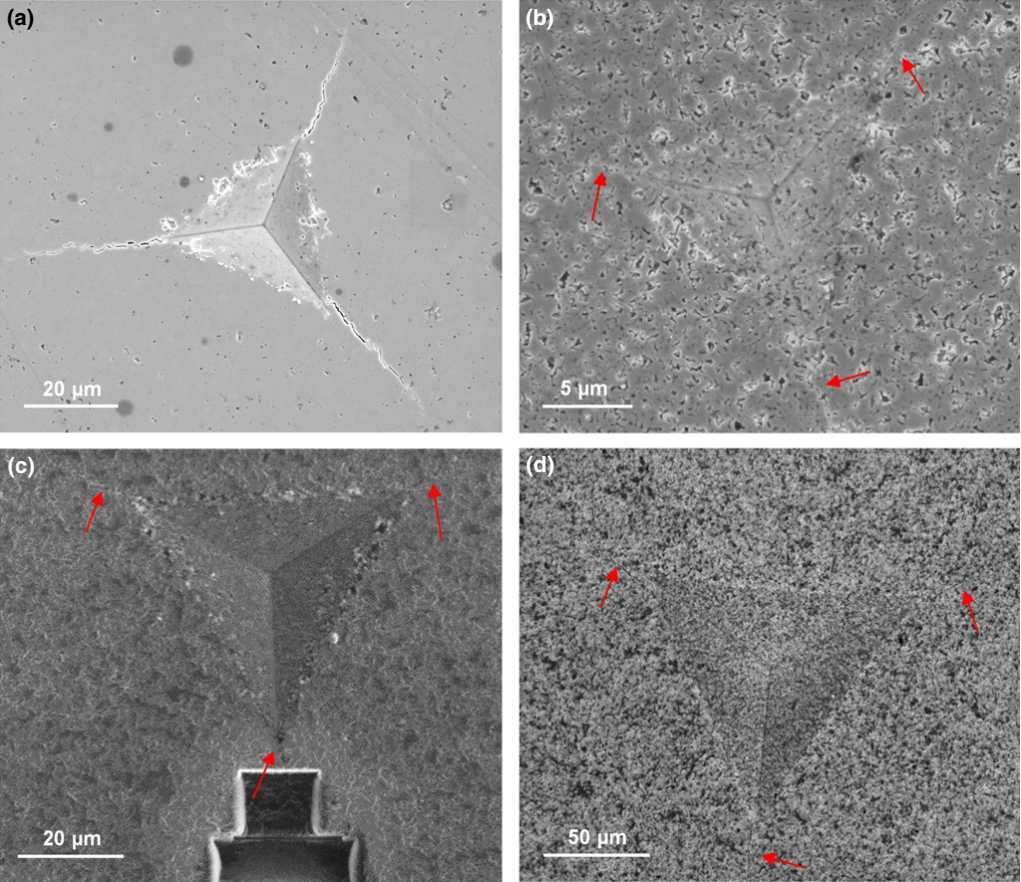
SEM micrographs showing radial cracks around the imprints of bulk specimens after sintering and indentation at different load (a) 1200°C and 5 N, (b) 1100°C and 500 mN, (c) 1000°C and 2 N, and (d) 900°C and 20 N. Note that the trenches in (c) were machined by FIB.

Accurate fracture toughness measurements require well‐defined indentation‐induced cracks.^29^ Clear cracks are observed in Fig. 1(a) in the 5.22% porosity specimen (sintered at 1200°C) after indentation at 5 N load. While crack morphologies generated by indentation at various loads for the specimens sintered at 1100°C and below are less clear to see due to their porous surfaces and the crack width being comparable to the pore/particle sizes. Nevertheless, the cracks were all identified and dimensions measured with the aid of high‐resolution SEM as arrowed in Figs. 1(b)–(d).

**Figure 2:**
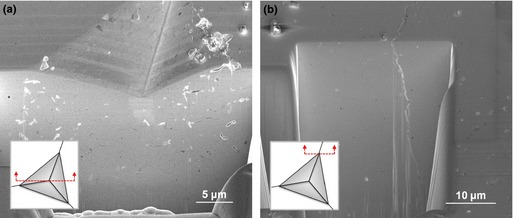
FIB‐sectioned features under the indented area after indentation at 5 N of a bulk specimen sintered at 1200°C: (a) cross section through the imprint center, (b) cross section through the imprint corner, showing a well‐developed crack.

In this study, the FIB/SEM was used to investigate the cross‐sectional cracking geometry. Two locations across one of the radial diagonals of the imprint in the specimen sintered at 1200°C were chosen for sequential FIB sectioning and SEM imaging, as presented in Fig. 2.

It can be seen from the above SEM images that cracking is not observed immediately below the contact area of the residual imprint. The sectioned face in Fig. 2(b) confirms the generation of the well‐developed radial crack (vertical orientation and horizontal propagation) from the corner of the residual imprint. The cracks penetrated under the specimen surface to a depth of several times the indentation depth without extending into or beneath the deformed zone for joining. Based on the above observations, the crack shape induced by Berkovich indentation here is categorized as the Palmqvist radial crack type,^30^ as the cracks generated from each extremity of the corner are independent, rather than joined to form the median or half‐penny shaped cracks beneath the deformed zone.^22^

**Figure 3:**
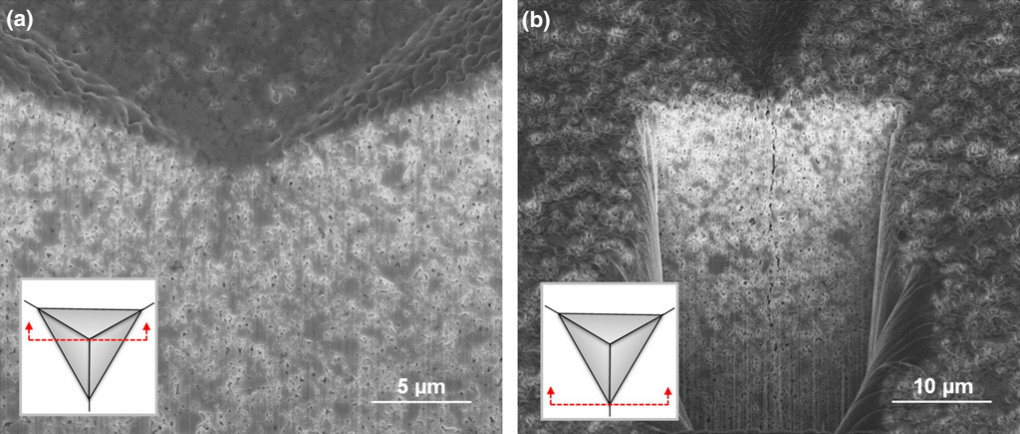
Micrographs of the crack after indentation at 2 N of a bulk specimen sintered at 1000°C: (a) cross section through the imprint center, (b) cross section through the imprint corner, showing a well‐developed crack.

Figure 3 shows the SEM images of the crack morphologies after indentation at 2 N for a specimen with intermediate porosity sintered at 1000°C. FIB sequential sectioning was also used to obtain cross‐sectional views, revealing the possible Palmqvist radial crack type as observed for the other samples in this study. However, it is not possible to identify whether the crack is transgranular or intergranular based on the micrographs obtained. An important feature in this specimen is that less densification is found in the deformed regions [Fig. 3(a)], as compared with the clear densification‐induced porosity gradient found in the indented films on dense substrates.^12^ This will be returned to in the later discussion.

#### 3.2.2 Comparison of Fracture Toughness Derived from Indentation and SEVNB

**Figure 4:**
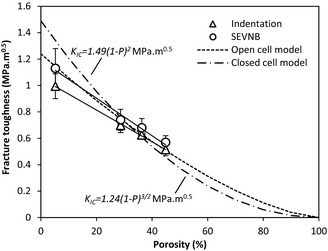
Indentation fracture toughness measured using indentation and SEVNB for bulk specimens as a function of porosity and their linear fits, compared with the two models.

Based on the elastic modulus, hardness and the crack dimensions measured the fracture toughness of the four types of bulk specimens were calculated using Eq. (2) and are compared to the SEVNB results as a function of porosity, as shown in Fig. 4.

It can be seen that the indentation results agree well with the SEVNB results to within experimental error, although the latter are consistently slightly higher than the former. This could be caused by the notch tips not being sufficiently sharp, and this tends to overestimate the toughness.^31^ The low toughness values for specimens sintered at temperatures below 1200°C are due to the high porosities of the specimens (>28%). The resulting toughness data for the specimens sintered at 1200°C (0.99 ± 0.10 MPa·m^1/2^ by indentation and 1.13 ± 0.15 MPa·m^1/2^ by SEVNB) are also close to the literature values reported by Chou *et al*.^9^ (1.10 ± 0.05 MPa·m^1/2^) and Huang *et al*.^32^ (0.91 ± 0.05 MPa·m^1/2^), as summarized in Table 3. Note that as shown in the table, a relatively large toughness was reported by Li *et al*.^33^ (1.75 ± 0.25 MPa·m^1/2^).

**Table 3:** Comparison of Fracture Toughness Measurements for Nominally Dense LSCF Specimens

Reference	Sintering conditions (in air)	Relative density (%)	Grain size (μm)	Measurement technique	Fracture toughness at RT (MPa·m^1/2^)
Chou *et al*.^9^	1250°C/4 h/300°C/h	95.4 ± 0.2	2.9	Vickers indentation	1.10 ± 0.05
Huang *et al*.^32^	1200°C/4 h/300°C/h	96.6 ± 0.2	0.6 ± 0.2	Vickers indentation	0.91 ± 0.05
Li *et al*.^33^	1200°C/2 h/300°C/h	98.3	0.8	Vickers indentation	1.75 ± 0.25
This work	1200°C/4 h/300°C/h	94.78 ± 0.01	1.6	SEVNB 3‐point bending	1.13 ± 0.15
				Berkovich indentation	0.99 ± 0.10

Figure 4 also shows approximately linear relationships of toughness versus porosity within the range of porosity investigated. There exist some nonlinear empirical expressions about the porosity dependence of fracture toughness in the literature, such as *K*_Ic_ *= *(1 − *P*)^3/2^ *K*_IC0_ *,* 34, 35 *K*_Ic_ *= *(1−*P*)^2^ *K*_Ic0_ *,* 35 and *K*_Ic_ *= e* ^*−bP*^ *K*_Ic0_,^36^ with *K*_Ic0_ being zero porosity toughness of the specimen. Two typical *K*_Ic_‐*P* models are plotted in Fig. 4 to compare with the current data. It is found that *K*_Ic_ *= *(1−*P*)^3/2^ *K*_Ic0_ (the open‐cell model, with *K*_Ic0_ **=* *1.24 MPa·m^1/2^) matches better with the current data than the other one *K*_Ic_ *= *(1−*P*)^2^ *K*_Ic0_ (the closed‐cell model, with *K*_Ic0_ **=* *1.49 MPa·m^1/2^). However, it should be emphasized that there is barely any theoretical basis for these empirical relations, neither for the linear fits shown in this study.

### 3.3 Indentation Results and Residual Stresses for Films

#### 3.3.1 Microstructural Characterization

Fracture toughness measurements of porous films need acceptable indents that show radial cracks without interfacial delamination. In this study, SEM examinations showed that no detecta‐ble cracking was found at any load in the films sintered at 900°C–1100°C, either in the nanoindentation load range (0–500 mN) or beyond (500–3000 mN). Nevertheless, for the films sintered at 1200°C, clear cracks at loads ranging from 200 to 500 mN were found.

**Figure 5:**
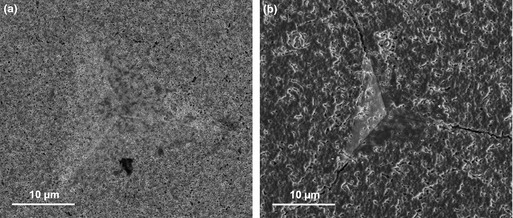
SEM micrographs of imprint morphologies after indentation at 500 mN for films sintered at (a) 1100°C, (b) 1200°C.

Examples of SEM images of the residual imprints after indentation at 500 mN for the films sintered at 1100°C and 1200°C are shown in Fig. 5.

**Figure 6:**
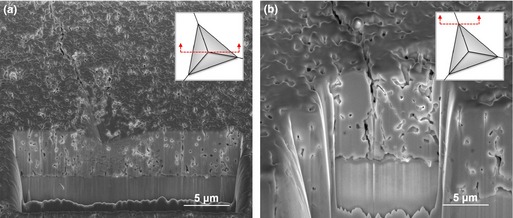
Micrographs of the FIB‐sectioned residual imprint: (a) cross section through the imprint center, showing densification underneath, (b) cross section through the imprint corner.

The above images show that Berkovich indentation in the highest density film produced well‐defined cracks without any chipping or secondary cracks. Sequential FIB sectioning and SEM imaging were also made for the films using to see the deformation below the residual imprint, as shown in Fig. 6.

Figure 6 shows no evident subsurface delamination. In Fig. 6(b), a radial crack propagated through the film thickness and arrested on the interface with the substrate. Therefore, the crack system in the indented film could be regarded as approximating a radial/Palmqvist crack.

#### 3.3.2 Film Fracture Toughness Derived from Indentation

**Figure 7:**
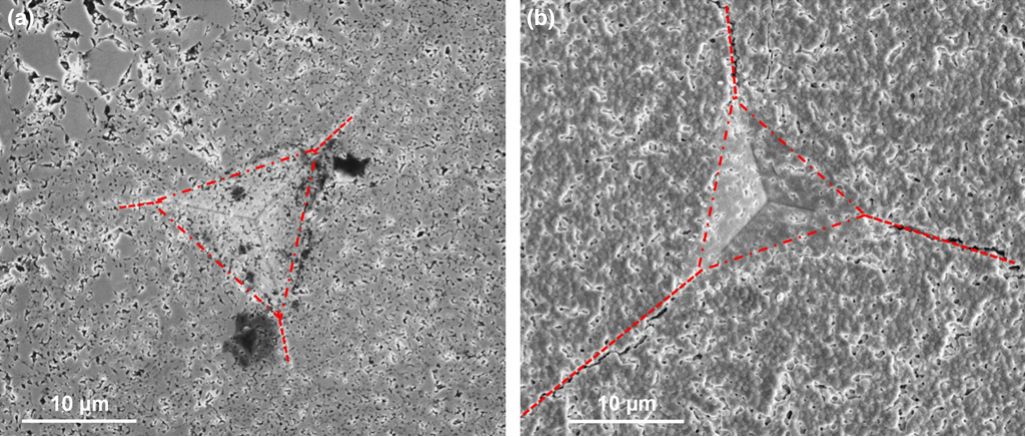
For a given load (500 mN), much longer cracks are found in a 1200**°**C sintered film (b) with 15% porosity, compared to 1100**°**C sintered bulk (a) with even much larger porosity (29%). Note that both images are at the same magnification and the indent sizes (triangles) are very close.

By applying Eq. (2), *K*_Ic_ of 0.17 ± 0.02 MPa·m^0.5^ was estimated for the film sintered at 1200°C (15.2% porosity). This value is very small and is much lower than that of the bulk specimens. According to Fig. 4 the toughness of a bulk specimen having the same porosity is expected to be ~0.9 MPa·m^0.5^. Figure 7 shows that for 500 mN indentation load, the film exhibited significantly longer cracks than a bulk specimen with even higher porosity.

#### 3.3.3 Residual Stresses in the As‐sintered Films

**Figure 8:**
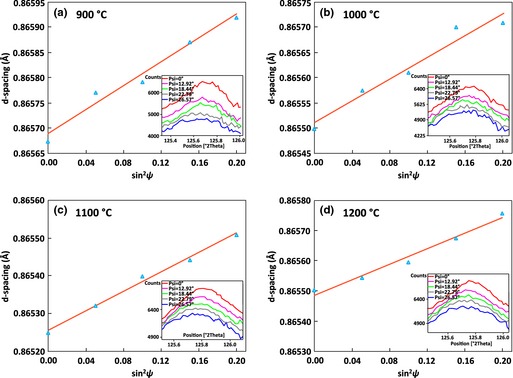
Plots of dψ as a function of sin^2^ψ for the different LSCF films based on the XRD patterns (insets) generated at (420) using sin^2^ψ method.

The interplanar *d*‐spacing *d*_ψ_ is plotted as a function of sin^2^ψ in Fig. 8 for the central diffraction peak at (420) plane based on the XRD patterns (insets) generated for the films sintered at different temperatures. The shifting of the diffraction peaks from high diffraction angles (2θ) to lower angles when ψ increased suggests the presence of tensile strains and stresses in all the films investigated. *d*_ψ_ versus sin^2^ψ shows good linearity. The resulting residual stresses for the films are listed in Table 4, showing increasing stress with higher sintering temperature as expected.

**Table 4:** Residual Stresses of the Films Determined by XRD

Sintering Temperature (°C)	900	1000	1100	1200
Residual stress measured (MPa)	59 ± 3	100 ± 2	179 ± 6	242 ± 10
Theoretical thermal mismatch stress (MPa)	79.4	131.9	270.9	399.7

The stresses induced by TEC mismatch in the films after cooling are estimated using Eq. (4) on an elastic regime basis.

(4) $$\sigma_{\mathrm{thermal}}=\frac{E}{1-\nu^{2}}\left(\alpha_{f}-\alpha_{s}\right)\left(T_{0}-T\right)$$

where α_f_ and α_s_ are TEC of LSCF and CGO, respectively. *E* and ν are the elastic modulus and Poisson's ratio of LSCF film, respectively. *T*_0_ is the stress free temperature, which is regarded the same as the sintering temperature in this study. *T* is the room temperature (25°C). The results are compared in Table 4 assuming no stress relief during cooling. Ideally, for each film, the thermal mismatch stress should equal the residual stress determined by XRD if there has been no stress relief during cooling. However, the thermal mismatch stress higher than the residual stress indicates that there has been some stress relief. When a larger TEC of LSCF (such as 24.5 × 10^−6^ K^−127^) is used for calculation, the theoretical stresses as well as the difference to the measured ones would increase greatly, suggesting that there could be much significant stress relief.

## 4 Discussion

Tensile residual stress in a brittle film can lead to an extensive network of cracks through the film; also known as channel cracking. The energy release rate for growth of a channel crack increases as the crack extends in the plane of the film and eventually reaches a constant value independent of crack length (steady‐state cracking) when the crack is longer than approximately a few times the film thickness. According to Beuth,^37^ the energy release rate *G*, due to steady‐state film cracking, can be calculated using the following Eq. (5),

(5) $$G=\frac{1}{2}\frac{\sigma^{2}\left(1-\nu^{2}\right)h}{E}\pi g$$

where σ is the measured residual stress, ν is the Poisson's ratio (assumed to be 0.3), *h* is the film thickness, *E* is the elastic modulus determined using nanoindentation, and *g* is a nondimensional parameter close to unity and depends on the relative elastic properties of the film and substrate. The calculated energy release rate and the resulting stress intensity for the onset of steady‐state cracking (using $K_{C}=\sqrt{\frac{E}{\left(1-\upsilon^{2}\right)}G}$ for plane strain) are presented in Table 5. The stress intensity is also compared with the fracture toughness for bulk material which had the same porosity as estimated based on the fits in Fig. 4.

**Table 5:** Strain Energy Release Rate and Stress Intensity for Channel Cracking Due to Residual Stress for the Films and Comparison with the Fracture Toughness for Bulk Material of Equivalent Porosity

Sintering Temperature (°C)	900	1000	1100	1200
*g*	0.86	0.90	1.03	1.11
Strain energy release rate (J/m^2^)	1.3 ± 0.1	2.7 ± 0.2	5.3 ± 0.1	7.7 ± 0.2
Stress intensity at RT (MPa·m^1/2^)	0.21 ± 0.01	0.36 ± 0.01	0.69 ± 0.01	0.96 ± 0.01
Fracture toughness for bulk material of equivalent porosity (MPa·m^1/2^)	0.49	0.59	0.77	0.90

The stress intensities for channel cracking in Table 5 are almost all lower than the fracture toughness of bulk material of equivalent porosity. Therefore, assuming that the fracture toughness of a film is similar to that of bulk material having the same porosity, these results are consistent with the observation that the as‐sintered films do not show networks of channel cracks when cooled to room temperature.

The results of this study raise two general issues regarding the application of indentation to measure the toughness of porous bulk materials and porous films on substrates. The first relates to the appropriateness of the analysis used for indentation of dense materials when applied to porous bulk materials. The present results show that the indentation toughness results are in good agreement with the SEVNB results for the porous bulk materials. This is at first unexpected because the permanent deformation mechanisms in dense and porous materials are different and some contribution from disruption of the particle networks and crushing densification might be expected under the indenter. Since this does not conserve volume, it would result in a smaller plastic zone for a given indenter depth and lower residual stresses on unloading. This would tend to lead to an overestimate of the toughness using Eq. (2) because a larger load would be required to give the same crack length. However, the FIB/SEM analysis under the indent shows no clear evidence of consolidation under the indenter. Furthermore, if anything the indentation toughness is consistently slightly smaller than the SEVNB toughness. Therefore, it is concluded that the permanent deformation in these porous materials does not involve significant consolidation and behaves macroscopically as if it were a dense effective material.

The second issue relates to the use of indentation to measure toughness for porous films on dense substrates. Despite the similar crack patterns exhibited in both LSCF bulk and films, it is worth noting that the toughness equation was originally developed for bulk materials and therefore its applicability for assessing film fracture toughness remains questionable^25^ even though it has been used to assess dense thin film fracture toughness by a number of researchers.^15^, ^38^ In the present work, we have found that indentation did not produce cracks in films sintered at 1100°C and below. This cannot be attributed to a low toughness. Furthermore, much longer cracks in films sintered at 1200°C compared with bulk specimen of similar porosity. It is therefore more likely that the standard analysis of the indentation method is not valid for these supported films. It might be expected that in general the toughness of a film on a substrate is inherently different from that of a bulk specimen of equal porosity.

There are issues in literature regarding the reliability of indentation for toughness measurement of brittle materials.^39^ However, our study shows that the toughness measured using indentation agrees well with that measured using SEVNB for bulk LSCF specimens (Fig. 4), suggesting the validity of the method for bulk specimens throughout the porosity range studied. Therefore, the different behavior of the films must be due to the substrate. According to Lawn *et al*.^40^ the tensile stresses provoking cracking are a combination of elastic stresses at peak load (responsible for the initial stages of cracking) and residual stresses during unloading (for the later stages of crack extension). (For the present films there are also the additional long‐range residual stresses measured due to TEC mismatch to be considered). During loading, the much stiffer substrate means that for a given load the stresses in the substrate will reduce the tensile (hoop) stresses in the film that initiate cracking. This will be more significant in the more porous films of lower modulus and it is suggested that in the films sintered at 1100°C and below, the combined indentation and TEC mismatch stresses in the film are insufficient to initiate the first stages of cracking. As a result, the residual stresses have no cracks to enlarge in the unloading phase and no cracks are produced. For the film sintered at 1200°C, the modulus is closer to that of the substrate and the stresses might become high enough to initiate cracks during loading. The stress intensity for channel cracking due to TEC mismatch is lower than the fracture toughness of the film and therefore these cracks do not propagate during loading. During unloading the cracks are subjected to the combined stress intensities of the indentation residual stress field and the long‐range TEC mismatch stress field and the two stress intensities are additive. For the observed crack length when the crack arrests in the film sintered at 1200°C, the stress intensity due to the indentation is 0.17 ± 0.02 MPa·m^1/2^ and the stress intensity due to the TEC mismatch stress is 0.96 ± 0.01 MPa·m^1/2^. The direct addition of these two values to account for the combined effects of indentation and TEC mismatch stresses for the same opening mode of loading could not seem appropriate, as the indentation also induce release of residual stress. Nevertheless, this analysis suggests that the real fracture toughness of the film is comparable to that of the bulk specimen having equivalent porosity (0.90 MPa·m^1/2^) and that the residual stress due to TEC mismatch is a major factor which caused a large underestimation of the toughness of this film as measured by applying the standard theory without considering the TEC mismatch stress.

## 5 Conclusions

This study presents the measurements of the room‐temperature fracture toughness of LSCF in both bulk and film forms using SEVNB 3‐point bending (for bulk) and Berkovich indentation (for bulk and films) after sintering at 900°C to 1200°C. The lengths of indentation‐induced cracks were estimated from SEM images of the indented surfaces. The FIB/SEM slice‐and‐view technique was employed to characterize the surface and subsurface crack morphologies, and confirmed the presence of radial Palmqvist crack systems induced by Berkovich indentation. The indentation toughness of bulk LSCF was determined to increase from 0.54 to 0.99 MPa·m^1/2^ as the sintering temperature increased from 900°C to 1200°C and the porosity decreased from 45% to 5%. The indentation toughness results were in good agreement with the SEVNB results (0.57 to 1.13 MPa·m^1/2^). In contrast, no indentation‐induced cracks were detected for porous films sintered at temperatures below 1200°C. XRD stress analysis showed that the films had a residual tensile stress due to the TEC mismatch between the film and the substrate. This stress was lower than the critical stress required to cause long‐range channel cracking in the films. The absence of indentation‐induced cracking in these films sintered at lower temperatures is suggested to be attributed to the fact that the substrate has much higher modulus than the films. The only observable cracks were found in the films sintered at 1200°C and resulted in an apparent toughness value of 0.17 MPa·m^1/2^ using the same theoretical treatment as used for the bulk specimens. This apparent toughness is much smaller than that of a bulk specimen of the same porosity. This apparently low value of toughness was found to be caused by the thermal expansion mismatch residual stress in the film as determined using XRD.
